# Discordance interpretation of left ventricular size between echocardiography and cardiac magnetic resonance in pediatric patients with aortic/mitral regurgitation

**DOI:** 10.1007/s10554-024-03073-3

**Published:** 2024-03-23

**Authors:** Anastasia Barros, Michelle Udine, Chris Spurney, Laura Olivieri, Yue-Hin Loke

**Affiliations:** 1 Nemours Children’s Health, Wilmington, DE USA; 2 Children’s National Hospital, Washington, DC USA; 3 Children’s Hospital of Pittsburgh of UPMC, Pittsburgh, PA USA

**Keywords:** Congenital heart disease, Mitral regurgitation, Aortic regurgitation, Pediatric cardiology, Cardiac magnetic resonance, Echocardiogram, Left ventricular end diastolic volume, Left ventricular internal diameter diastolic, Z-score

## Abstract

**Purpose:**

This study investigated discordance between echocardiography (echo) and cardiac magnetic resonance (CMR) measurements of the left ventricle (LV) in pediatric patients with aortic and/or mitral regurgitation (AR/MR).

**Methods:**

Retrospective cohort study of pediatric patients. The cohorts were comprised of patients with AR/MR vs. non-AR/MR. Left ventricular end diastolic volume (LVEDV) by CMR and left ventricular internal diameter diastolic (LVIDd) by echo were obtained from clinical reports then echo images were reviewed to remeasure LVEDV by bullet method. Left ventricular internal diameter systolic (LVIDs) and left ventricular ejection fraction (LVEF) measurements by echo and LVEF by CMR were obtained from clinical reports. Fractional shortening (FS%) was recalculated. Z-scores were calculated using normative data. Correlation between echo and CMR LV measurements was assessed using correlation coefficients. Bland-Altman plots assessed bias between imaging modalities. Receiver operator characteristic (ROC) analysis was performed for detection of LV enlargement and LV dysfunction.

**Results:**

AR/MR patients had greater discrepancy in LV size interpretation by Z-score compared to non-AR/MR patients. This discrepancy persisted when the bullet method short axis measurements were incorporated. There was negative bias in echo-based measurements compared to CMR. The diagnostic performance of echo in identifying moderate LV enlargement was worse for AR/MR pediatrics patients.

**Conclusion:**

The discordant interpretation of LV size by echo compared to CMR is worse in pediatric patients with AR/MR when compared to patients without AR/MR even when short axis measurements are incorporated. This finding suggests non-uniform geometrical changes in the LV as it enlarges due to AR/MR.

## Introduction

Interpretation of left ventricular (LV) size is essential to the management of congenital heart disease in pediatric patients. LV size is particularly important in patients with aortic and/or mitral regurgitation (AR/MR) which leads to LV enlargement. The size of the LV and rate of increase in that size have been shown to correlate with clinical outcomes. Increased LV size can relate to an increased rate of valve replacement, poor outcomes after valve replacement, increased left atrial size, increased rate of heart transplant and death [1–5]. This findings demonstrate the importance of LV size in the management of AR/MR such as in mitral valve prolapse, cleft of the mitral valve, AV canal defect, bicuspid aortic valve as well as a sequalae of surgical correction for congenital heart disease such as balloon angioplasty for aortic stenosis, Fontan circulation and the Ross procedure.

Cardiac magnetic resonance (CMR) provides a reproducible and accurate measurement of ventricular dimensions. This high level of reproducibility has been documented in previous research analyzing intra-observer, inter-observer and inter-examination errors. CMR has been shown to have higher reproducibility than any other form of cardiac imaging [6, 7]. Echocardiography (echo), however, is the front-line test for serial assessment of LV enlargement, including cases of AR/MR. Echo is more accessible than CMR due to its lower cost, wider availability, portability, and, especially in the case of children, is more easily tolerated [8, 9]. Echo assessment of LV size in pediatric patients either relies on linear left ventricular internal diameter diastolic (LVIDd) or five-sixth area length to calculate LVEDV (“bullet method”) while CMR relies on LVEDV [10, 11]. 

Large normative pediatric datasets have been developed to interpret LV size for echo and CMR. These studies were born out of the scarcity of normal data for children. The Pediatric Heart Network (PHN) dataset is a large multicenter cohort that provides normal data for echo parameters [12]. Published normative datasets for CMR parameters are mostly single center studies [13, 14]. The differences in interpretation of LV size between echo and CMR modalities have not been formally assessed in the pediatric population. Such discrepancy has been reported in the adult population [15]. The documented discrepancy between echo and CMR demonstrates the crucial importance of understanding inter-modality variation in LV size prior to making clinical decisions.

In this study, the discordance between echo and CMR measurements of the LV in pediatric patients with AR/MR was investigated using LVIDd and bullet method LVEDV by echo and LVEDV by CMR. This study is of importance given the lack of such research in the pediatric population and the clinical implications of a discrepancy between echo and CMR. We hypothesize that AR/MR patients will have a larger discordance in interpretation of LV enlargement between echo and CMR if only echo LVIDd is used and that the inclusion of a short axis measurement through the LVEDV by bullet method will decrease the level of discordance.

## Methods

This was a retrospective cohort study conducted on pediatric patients aged 18 years and younger with echo and CMR studies completed at Children’s National Hospital between the years of 2014 and 2022. The study was approved by the Children’s National Hospital institution review board. The study was composed of two retrospective cohorts, one with patients diagnosed with AR/MR and one without AR/MR, which served as the control.

### Inclusion criteria

Selected patients for the AR/MR group were determined by searching the CMR database using the terms aortic regurgitation and mitral regurgitation. The non-AR/MR cohort was comprised of pediatric patients with diagnoses of sickle cell disease, cardiomyopathy, heart transplant and rule out for myocarditis. Patients were included in the study if they had an echo within one year of a CMR, the studies were of good diagnostic quality and age of patient was 18 years or younger at the time the imaging was done. Specifically for the AR/MR cohort, patients were included if CMR showed the presence of greater than or equal to mild AR/MR, defined as regurgitant fraction ≥ 5%. Inclusion criteria was based on the CMR regurgitant fraction given echo provides only qualitative assessments of valve regurgitation. A regurgitant fraction of ≥ 5% is used as the lab standard for ≥ mild AR/MR at Children’s National Hospital.

### Exclusion criteria

Patients were excluded if the imaging studies were of poor diagnostic quality or there was no echo within one year of the CMR. Patients were excluded from the final AR/MR group if the echo report stated no AR/MR or the AR/MR was trivial.

### Echo measurements

All echo studies were performed on either a Philips IE-33/Epiq (Philips, Cambridge, MA, USA), or GE Vivid E-95 (General Electric, Boston, MA, USA), following standard methodology according to the American Society of Echocardiography [16]. All measurements were performed on IntelliSpace Cardiovascular workspace.

**Fig. 1:**
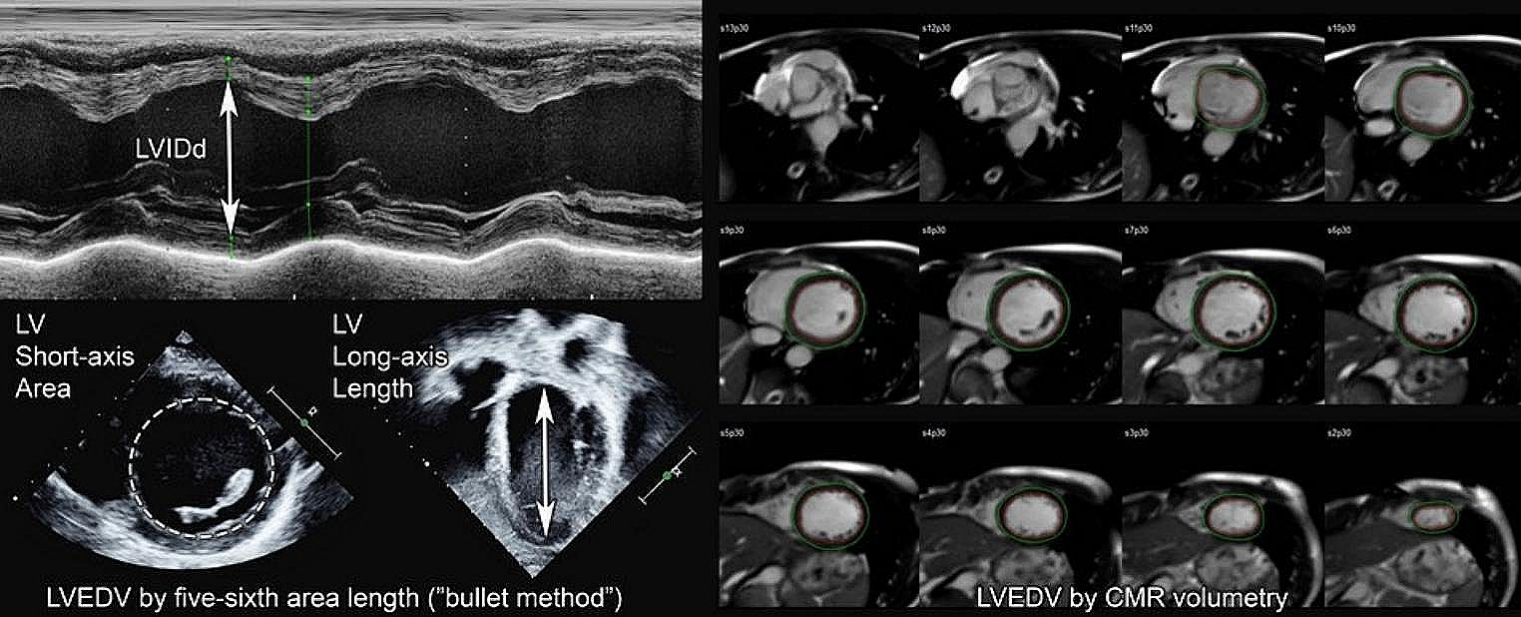
Representative images of left ventricle measurements by M mode LVIDd, bullet method LVEDV and CMR LVEDV

LVIDd measurements by echo were obtained from clinical reports as depicted in Fig. 1. The PHN normative dataset was used to calculate Z-scores for all echo data. PHN involved 19 centers and is comprised of 3215 patients, age < 1 month to 18 years [12]. For the AR/MR cohort, echo imaging was then independently reviewed by a single observer to remeasure LVEDV using the bullet method as depicted in Fig. 1. Z-scores were calculated using the PHN dataset. LVEDV using the Simpson method was not utilized as this method was not incorporated into the PHN dataset, and thus Z-scores of LVEDV by Simpsons could not be feasibly generated. Left ventricular internal diameter systolic (LVIDs) and left ventricular ejection fraction (LVEF) measurements by echo were obtained from clinical reports. Fractional shortening (FS%) was calculated using LVIDd and LVIDs.

### CMR measurements

All CMR studies were performed on a 1.5 S Aera. LVEDV measurements by CMR were obtained from clinical reports as depicted in Fig. 1. Z-scores were re-calculated using normative data. The Buechel et al. and Olivieri et al. datasets were used for CMR Z-scores based on age. The Olivieri et al. database was used to calculate CMR Z-scores for patients aged 12 years and younger. This normative dataset included 149 patients, age 22 days to 12 years [13]. Buechel et al. was used for patients over age 13 years. That data set is comprised of 50 patients, age 7 months to 18 years [14]. The decision to use two CMR normative data sets was based on the age of patients included in each data set. CMR Z-scores were then recalculated using the PHN data set. LVEF measurements by CMR were obtained from clinical reports. Moderate LV enlargement was defined as LVEDV Z-score > 3. LV dysfunction was defined as LVEF < 55%.

### Statistical analysis

Correlation coefficients were used to assess the correlation between echo and CMR measurements of LV size using various ways of measuring the LV and methods of standardizing those measurements. Comparisons included echo LVIDd to CMR LVEDV and echo LVEDV by bullet method to CMR LVEDV. This analysis was first completed using the conventional Z-scores [12–14]. The analysis was then redone for the AR/MR group using PHN Z-scores for both echo and CMR to assess for improvement in correlation [12]. Bland-Altman plots were created using both conventional and adjusted Z-scores. Receiver operative characteristic (ROC) analysis was performed for detection of moderate LV enlargement defined as CMR LVEDV Z-score > 3. Correlation coefficients were also used to assess the correlation between echo FS% and CMR LVEF as well as echo LVEF to CMR LVEF. This comparison was done using raw values. ROC analysis was performed for detection of LV dysfunction defined as LVEF < 55%.

## Results

### Demographics

A total of 343 pediatric patients were included in the final analysis with 81 in the AR/MR cohort and 247 in the non-AR/MR cohort. A total of 10 patients were excluded due to lack of adequate echo imaging of the LV short-axis for bullet method measurements. All patients had diagnostic quality CMR images. The characteristics of each cohort as well as baseline echo data are summarized in Tables 1 and 2. There was no statistically significant difference between the two cohorts in terms of age, gender or BSA. The indexed LVEDV was larger in the AR/MR group compared to non-AR/MR (*p* < 0.0001). There was a greater percentage of patients with moderate LV enlargement in the AR/MR group than the non-AR/MR group, while the non-AR/MR group had a larger portion of LV dysfunction present.

**Table 1:** Patient information by cohort

	AR/MR (*n* = 81)	Non-AR/MR (*n* = 247)	p-value
Age (years)	11.6 ± 5.0	12.4 ± 4.4	NS
Gender (% female)	34 (42% female)	112 (42% female)	NS
BSA (m [2])	1.3 ± 0.5	1.4 ± 0.5	NS
LVEDVi (mL/m^2^)	113 ± 35	87 ± 36	< 0.0001
LV Dysfunction	14 (18%)	115 (44%)	< 0.0001
Moderate LV enlargement	32 (39%)	30 (11%)	< 0.0001

**Table 2:** Echo measurements by cohort

	AR/MR (*n* = 81)	Non-AR/MR (*n* = 247)	p-value
LVIDd (cm)	5.25 ± 1.10	4.54 ± 0.87	< 0.0001
IVSd (cm)	0.82 ± 0.21	0.97 ± 0.26	< 0.0001
LVPWd (cm)	0.79 ± 0.21	0.77 ± 0.36	NS
LVIDs (cm)	3.35 ± 0.80	3.04 ± 0.81	0.0029
IVSs (cm)	1.12 ± 0.28	1.06 ± 0.30	NS
LVPWs (cm)	1.30 ± 0.32	1.19 ± 0.31	0.0063
FS (%)	36 ± 6	33 ± 8	0.0021
LVEF (%)	63 ± 6	59 ± 10	0.008

### Agreement in conventional Z-scores between echo and CMR

**Fig. 2:**
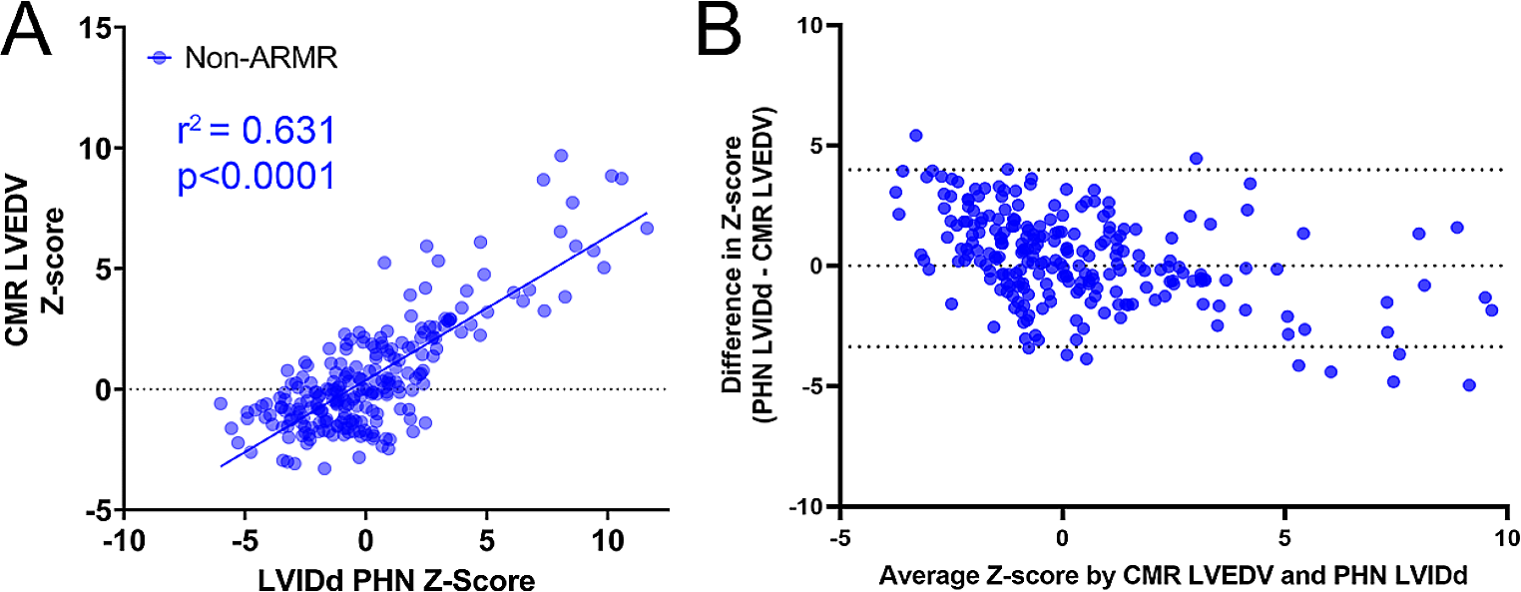
Correlation between echo LVIDd and CMR LVEDV Z-scores in non-AR/MR group

**Fig. 3:**
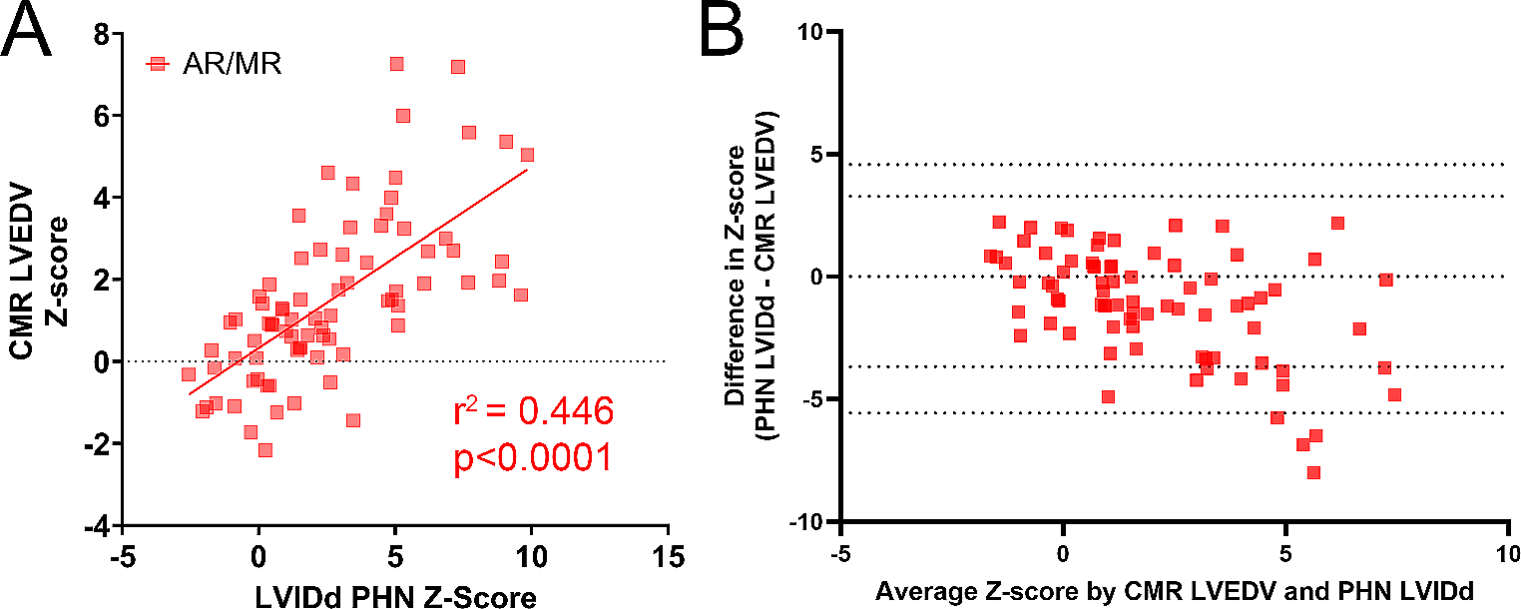
Correlation between echo LVIDd and CMR LVEDV Z-scores in AR/MR group

**Fig. 4:**
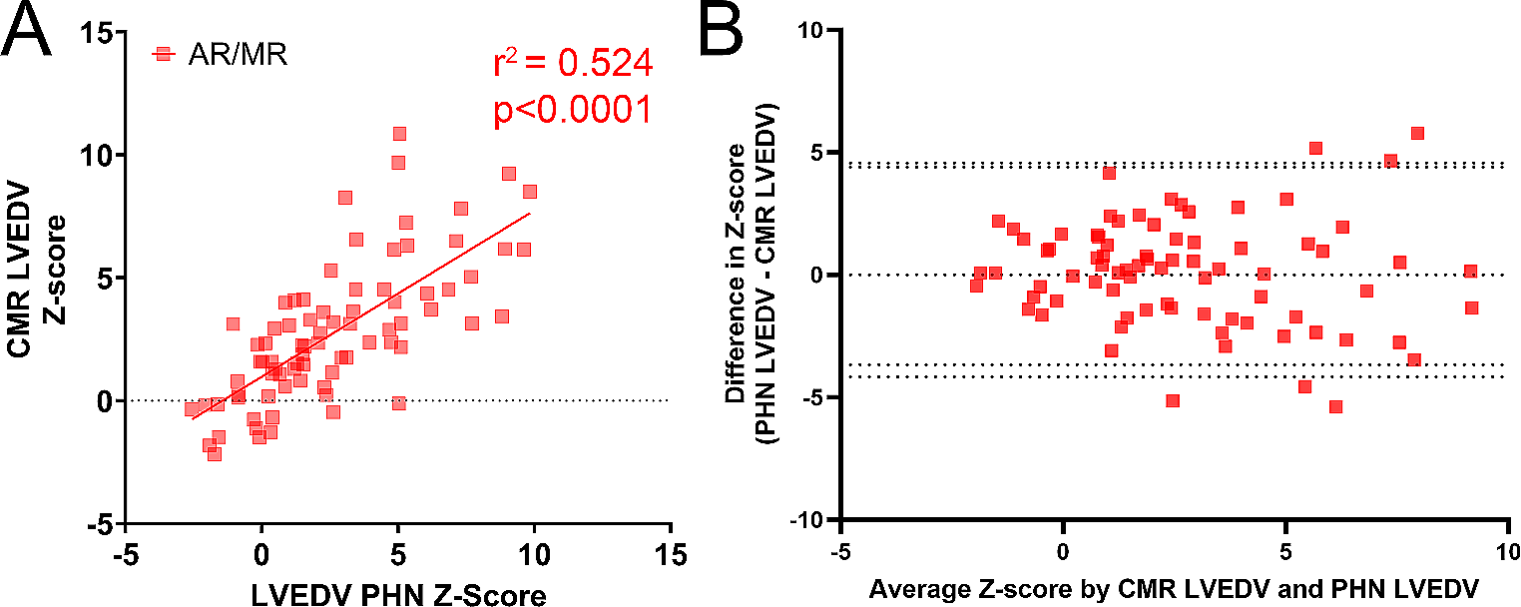
Correlation between echo bullet LVEDV and CMR LVEDV Z-scores in AR/MR group

Scatter plots with respective correlation coefficients for each comparison are summarized in Figs. 2, 3 and 4. AR/MR patients had lower correlation between echo LVIDd and CMR LVEDV Z-scores compared to non-AR/MR patients (r^2^ = 0.446 vs. r^2^ = 0.631, *p* = 0.016 – Figs. 2A and 3A respectively). The correlation improved in the AR/MR group when LVEDV by bullet method was used as the echo measurement, with a correlation coefficient of r^2^ = 0.524. The Bland-Altman for the non-AR/MR group had a bias of 0.3151 ± 1.875 (Fig. 2B). For the AR/MR group, when using LVIDd, it had a bias of -1.144 ± 2.255 (Fig. 3B) and for LVEDV had a bias of 0.1177 ± 2.184 (Fig. 4B).

### Agreement in adjusted Z-scores between echo and CMR in AR/MR group

**Fig. 5:**
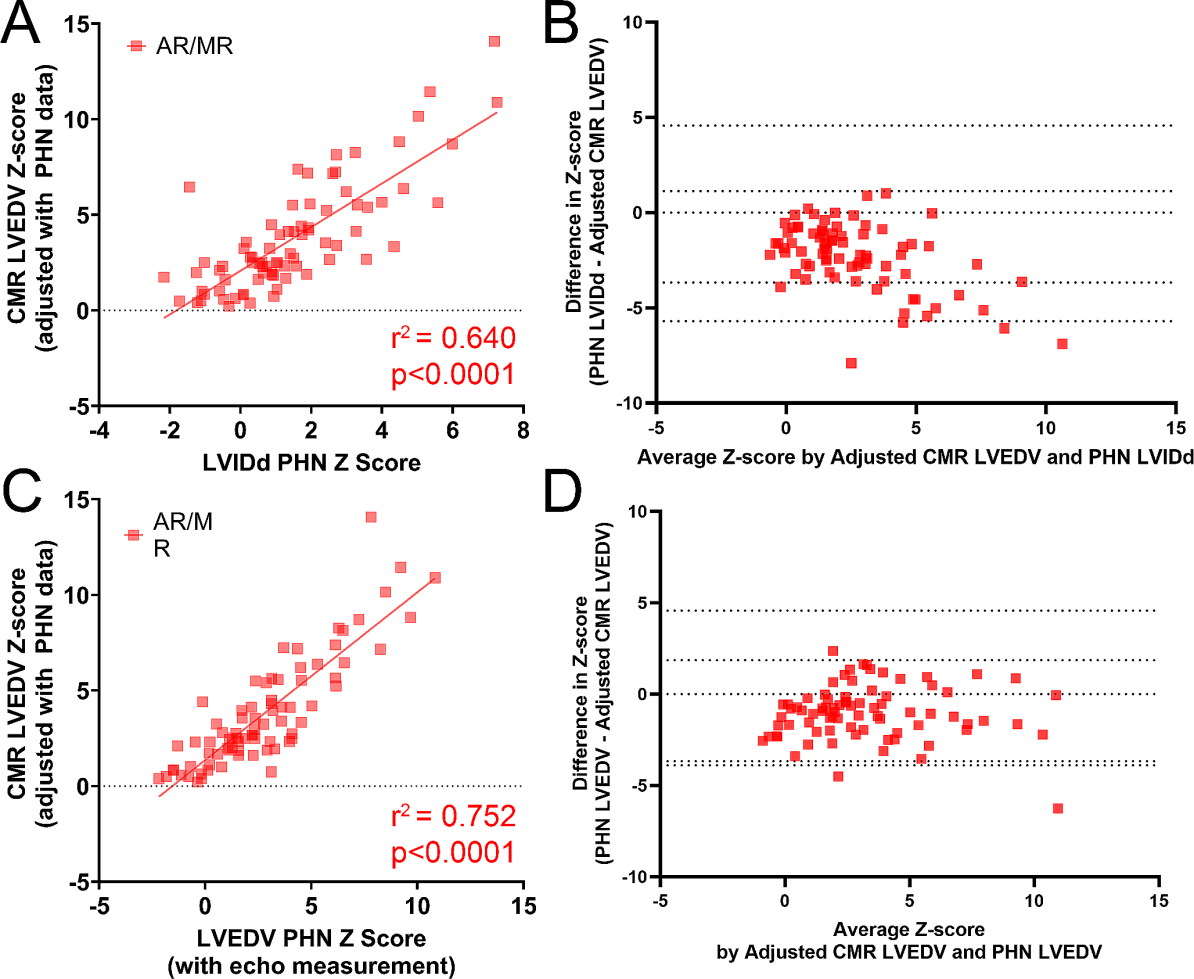
Correlation between echo LVIDd and bullet LVEDV with CMR LVEDV PHN adjusted Z-scores in AR/MR group

Scatter plots with respective correlation coefficient for each comparison are summarized in Fig. 5. The correlation between echo and CMR measurements in the AR/MR group improved when utilizing PHN normative data for both echo and CMR Z-scores, with a correlation coefficient r^2^ = 0.640 between LVIDd and CMR LVEDV, and r^2^ = 0.752 between echo LVEDV and CMR LVEDV. Bland-Altman analysis demonstrated negative bias for both LVIDd (-2.281 ± 1.742) and LVEDV by Bullet (-1.019 ± 1.467).

### Detection of LV enlargement

**Fig. 6:**
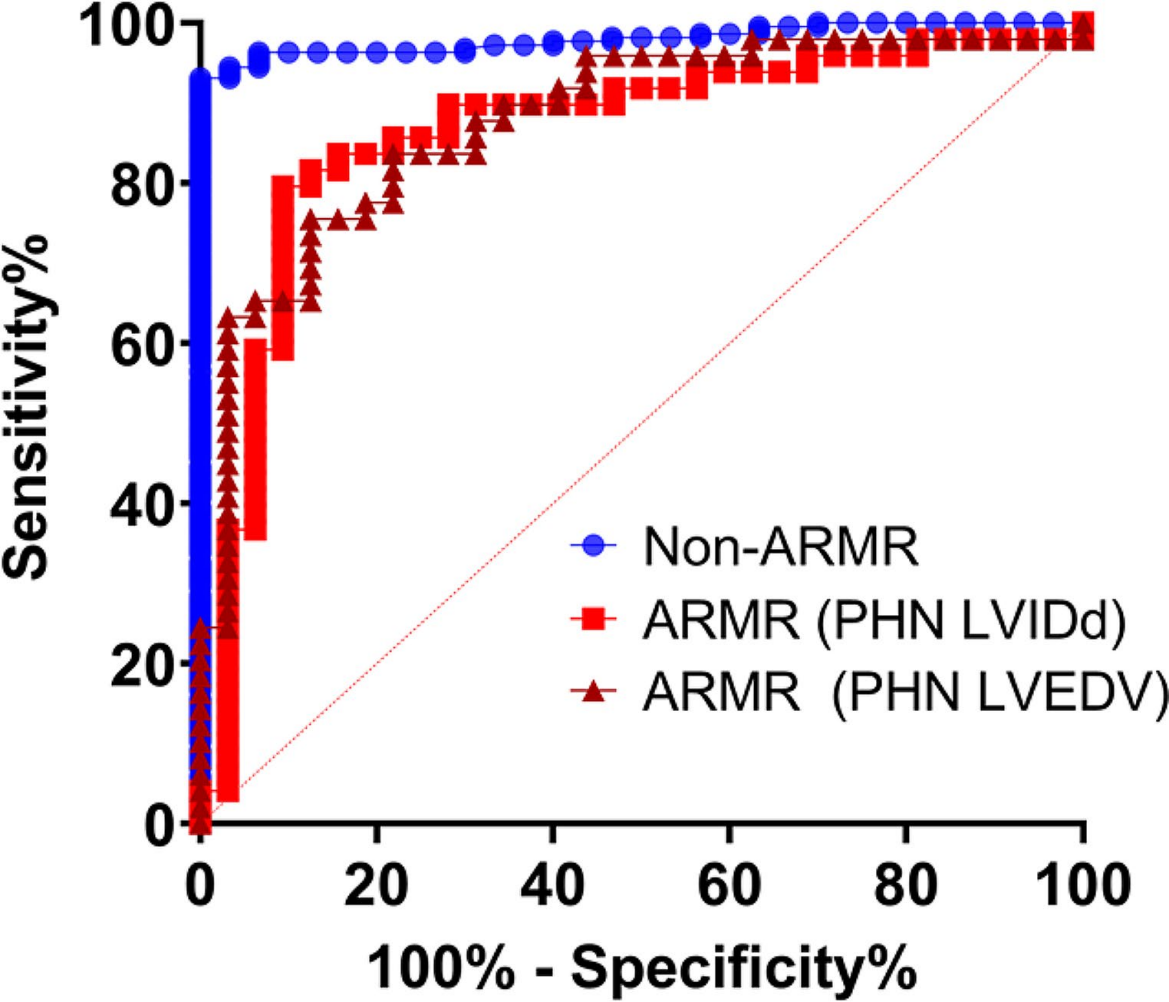
Detection of moderate LV enlargement by cohort and LV measurement

Next step of analysis used ROC curves for detection of moderate LV enlargement defined as CMR LVEDV Z-score > 3 as summarized in Fig. 6. With both LVIDd and LVEDV bullet method, AR/MR patients had lower AUC when compared to non-AR/MR. For LVIDd, AR/MR AUC = 0.863 vs. non-AR/MR AUC = 0.980, with *p* = 0.004. Then AR/MR LVEDV by bullet method AUC = 0.878 vs. non-AR/MR AUC = 0.980, with *p* = 0.008.

### Agreement in LV systolic function between echo and CMR

**Fig. 7:**
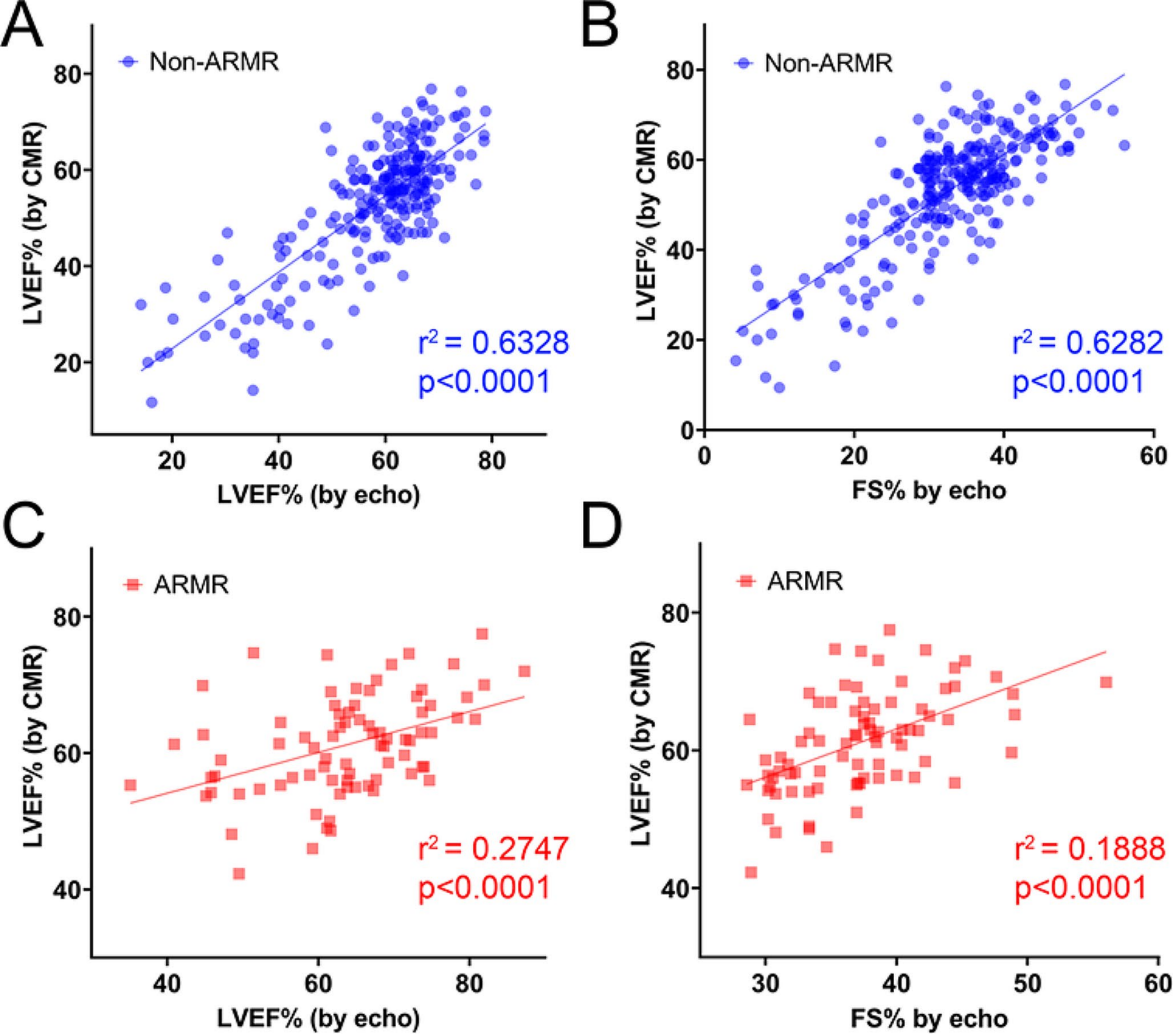
Correlation between echo LVEF% and FS% with CMR LVEF%

Scatter plots with respective correlation coefficient for each comparison are summarized in Fig. 7. For the non-AR/MR group, echo LVEF had correlation with CMR LVEF of r^2^ = 0.6328, *p* < 0.0001 and echo LVEF to CMR FS% of r^2^ = 0.6282, *p* < 0.0001. For the AR/MR group, echo LVEF was moderately correlated with CMR LVEF with r^2^ = 0.2747, *p* < 0.0001. FS% was moderately correlated with CMR LVEF with r^2^ = 0.1888, *p* < 0.0001).

### Detection of LV dysfunction

**Fig. 8:**
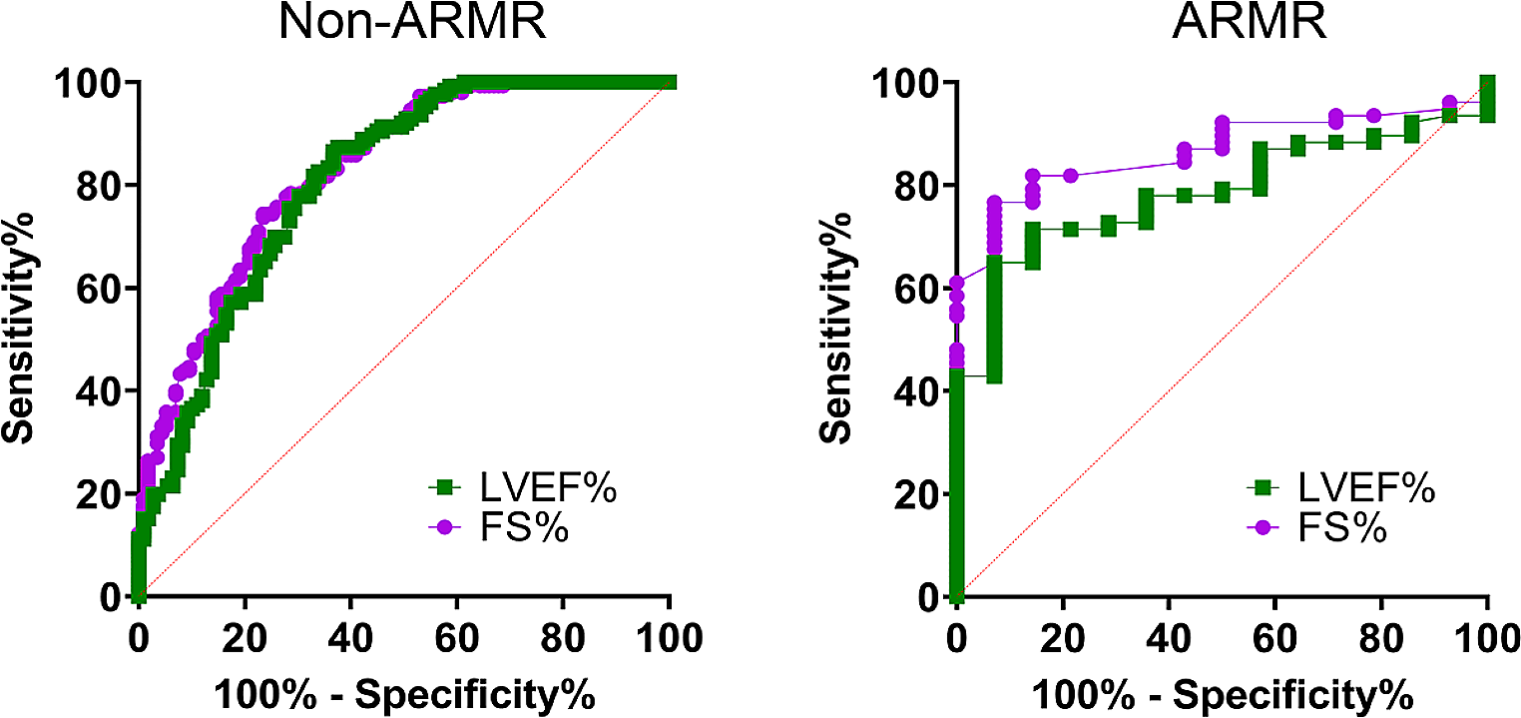
Detection of LV dysfunction by cohort and functional measurement

The last step of analysis used receiver operator characteristic curves for detection of LV dysfunction defined as LVEF < 55% as summarized in Fig. 8. With both LVEF and FS%, AR/MR patients had no significant differences in AUC when compared to non-AR/MR. For LVEF AUC = 0.8069 ± 0.02859 vs. AUC = 0.7843 ± 0.05076, respectively with *p* = 0.716 and then FS% AUC = 0.8278 ± 0.02516 vs. AUC = 0.8660 ± 0.03869, respectively with *p* = 0.43.

## Discussion

This study aimed to analyze discrepancies between echo and CMR measurements of the LV in pediatric patients with AR/MR. Our study found that pediatric patients with AR/MR had a greater discrepancy in LV size interpretation by Z-score when compared to non-AR/MR patients. This discrepancy persisted when short axis measurements were incorporated to derive LVEDV by bullet method.

Given the significant range of patient size in pediatric cardiology, Z-scores are often used to serially interpret measurements to determine the development of ventricular enlargement and dysfunction [17]. Provided an adequately representative normative dataset and a patient with a normal body surface area, Z-scores can provide LV size interpretation representative of a pathological process such as AR/MR by identifying those outside the “normal” range of standard deviation [12–14, 17]. If a patient is at an extreme end of body size the Z-scores may under or overestimate the measurement [17]. At the same time, the interchanging use of normative datasets depending on imaging modality may lead to discrepancies. Limitations still persist even when applying the same normative equations on both echo and CMR measurements as demonstrated in Fig. 5; even though Z-scores are more concordant, there is still a consistent negative bias such that echo-based measurements would produce lower Z-scores compared to CMR measurements of the same patient.

The noted discrepancies of LV interpretation are likely the result of non-uniform changes in LV geometry that may be less sensitive to echo-based measurements. In AR, the LV has been shown to have a greater curvature of the anterobasal/anterolateral/inferoapical regions and lower curvature in the anteroapical region resulting in a round shape [18]. In MR, LV enlargement also develops into a spherical shape leading to worsening remodeling [19]. These factors likely contribute to the differences in interpretation by LVIDd or LVEDV by bullet which rely on geometrical assumptions that the LV is conical or bullet shaped. This dimensional limitation can also be seen in functional assessments in the LV, as noted in a study by Clark et al. where there was only 64.4% agreement between echo and CMR regarding LVEF [15]. 

In our study the overall diagnostic performance of echo in identifying moderate LV enlargement was worse for AR/MR pediatrics patients compared to controls. These discrepancies between echo and CMR may have significant ramifications to the long-term management of AR/MR as LV measurements often play a crucial aspect in determining intervention. In adult cohort studies such as Malahfji et al., indexed LVEDV and LVESV by CMR were independently associated with the composite adverse outcome of development of symptoms, LV dysfunction or death and volumetric measurements demonstrated favorable performance over echo-based LV diameters in guiding aortic valve replacement therapy [20]. However, as evidenced by a survey conducted by Boyett Anderson and Hokasen, there is significant practice variation amongst pediatric cardiology providers in terms of follow-up and intervention of children with AR [21]. In the pediatric population, children are likely followed serially from a young age by linear echo measurements and may then be found to have drastic re-interpretation of LV size after an evaluation by CMR leading to a potential significant change in the provider’s approach. The picture is further complicated by the lack of standard cut off thresholds for intervention based on LV size. Future research is needed to determine standards regarding LV size in relation to intervention on AR/MR and such work must include both echo and CMR due to the clear discrepancy between the two methods.

While CMR remains an accurate standard for absolute LV measurements, it does not have the robust normative studies in the same scope as the PHN dataset. Olivieri et al. and Buechel et al. encompass two single center studies with approximately 150 normal controls, compared to the multicenter nature of PHN encompassing 3000 studies [12–14]. This study practically demonstrates how differences in the regression model between the datasets contribute to the discrepancies in interpretation. Even if there are attempts to apply the same regression equation between imaging modalities there is consistently a negative bias in echo-based measurements, likely from the aforementioned limitations in geometrical assumptions. Thus, for pediatric cardiologists there needs to be continued attention to the source and derivation of Z-scores, given its emphasis in managing pediatric heart disease.

The specific limitations within our study include the following: Only the LVEDV measurements by bullet method were all measured by a single observer, whereas the LVEDV measurements by CMR were directly taken from clinical reports, potentially risking an effect from interobserver variability. This effect is likely minimal as the three readers responsible for CMR reports during the study period have previously demonstrated excellent agreement for LVEDV (average intraclass correlation coefficient of 0.994 for LVEDV) [13]. Secondly, was the reliance on single center normative data sets for CMR Z-scores. We also did not exclude patients with abnormally low or high body surface area such as small infants which may have potentially skewed the Z-score derivation.

Further research is needed to investigate three dimensional measurements that account for the increasingly spherical shape of the LV in patients with AR/MR in order to improve concordance in interpretation of LV enlargement between echo and CMR. As suggested by Clark et al., the concordance between echo and CMR may improve if three-dimensional echo was measurements are used [15]. Additional work also needs to be done in the development of large, multicenter normative datasets for CMR in order to improve Z-score calculations. Continued comparisons in the performance of different Z-scores, both for echo and CMR is also important.

## Conclusion

The discordant interpretation of LV size by echo compared to CMR is worse in pediatric patients with AR/MR when compared to patients without AR/MR even when short axis measurements are incorporated. This finding suggests non-uniform geometrical changes in the LV as it enlarges due to AR/MR.

## Data Availability

No datasets were generated or analysed during the current study.

## References

[1] Barbieri A, Giubertoni E, Bartolacelli Y, Bursi F, Manicardi M, Boriani G (2018). New classification of geometric patterns considering left ventricular volume in patients with chronic aortic valve regurgitation: prevalence and association with adverse cardiovascular outcomes. Echocardiography.

[2] Schott JP, Dixon SR, Goldstein JA (2021). Disparate impact of severe aortic and mitral regurgitation on left ventricular dilation. Catheter Cardiovasc Interv off J Soc Card Angiogr Interv.

[3] Ghelani SJ, Lu M, Sleeper LA (2022). Longitudinal changes in ventricular size and function are associated with death and transplantation late after the Fontan operation. J Cardiovasc Magn Reson.

[4] Rusinaru D, Tribouilloy C, Grigioni F (2011). Left atrial size is a potent predictor of Mortality in Mitral Regurgitation due to Flail leaflets: results from a large International Multicenter Study. Circ Cardiovasc Imaging.

[5] Butcher SC, Pio SM, Kong WKF et al (2022) Left ventricular remodelling in bicuspid aortic valve disease. *Eur Heart J Cardiovasc Imaging* ;23(12):1669–1679. 10.1093/ehjci/jeab284. PMID: 34966913 10.1093/ehjci/jeab284 34966913

[6] Pattynama P, Lamb H, van der Velde E, van der Wall E, de Roos A (1993). Left ventricular measurements with cine and spin-echo MR imaging: a study of reproducibility with variance component analysis. Radiology.

[7] Grothues F, Smith GC, Moon JCC et al (2002) Comparison of Interstudy Reproducibility of Cardiovascular Magnetic Resonance With Two-Dimensional Echocardiography in Normal Subjects and in Patients With Heart Failure or Left Ventricular Hypertrophy. *Am J Cardiol* ;90(1):29–34. 10.1016/s0002-9149(02)02381-0. PMID: 12088775 10.1016/s0002-9149(02)02381-0 12088775

[8] Hudson DM, Heales C, Meertens R (2022). Review of claustrophobia incidence in MRI: a service evaluation of current rates across a multi-centre service. Radiography.

[9] Dong S, Zhu M, Bulas D (2019). Techniques for minimizing sedation in pediatric MRI. J Magn Reson Imaging.

[10] Lopez L (2010) Recommendations for quantification methods during the performance of a Pediatric Echocardiogram: a Report from the Pediatric Measurements Writing Group of the American Society of Echocardiography Pediatric and Congenital Heart Disease Council. J Am Soc Echocardiogr. ;23(5) 10.1016/j.echo.2010.03.019 20451803

[11] Fratz S, Chung T, Greil GF et al Guidelines and protocols for cardiovascular magnetic resonance in children and adults with congenital heart disease: SCMR expert consensus group on congenital heart disease. Published online 2013. 10.1186/1532-429X-15-51 PMC3686659 23763839

[12] Lopez L, Colan S, Stylianou M (2017). Relationship of echocardiographic Z scores adjusted for body surface area to Age, Sex, Race, and ethnicity: the Pediatric Heart Network Normal Echocardiogram Database. Circ Cardiovasc Imaging.

[13] Olivieri LJ, Jiang J, Hamann K (2020). Normal right and left ventricular volumes prospectively obtained from cardiovascular magnetic resonance in awake, healthy, 0–12 year old children. J Cardiovasc Magn Reson.

[14] Buechel EV, Kaiser T, Jackson C, Schmitz A, Kellenberger CJ (2009). Normal right- and left ventricular volumes and myocardial mass in children measured by steady state free precession cardiovascular magnetic resonance. J Cardiovasc Magn Reson.

[15] Clark J, Ionescu A, Chahal CAA (2023). Interchangeability in Left Ventricular Ejection Fraction measured by Echocardiography and cardiovascular magnetic resonance: not a Perfect Match in the Real World. Curr Probl Cardiol.

[16] Lopez L, Saurers DL, Barker PCA (2024). Guidelines for performing a Comprehensive Pediatric Transthoracic Echocardiogram: recommendations from the American Society of Echocardiography. J Am Soc Echocardiogr.

[17] Chubb H, Simpson JM (2012) The use of Z-scores in paediatric cardiology. Ann Pediatr Cardiol. ;5(2) 10.4103/0974-2069.99622 PMC3487208 23129909

[18] Barletta G, Di Donato M, Baroni M, Fantini A, Fantini F (1993). Left ventricular remodeling in chronic aortic regurgitation. Int J Card Imaging.

[19] Athanasuleas CL, Stanley AWH, Buckberg GD (2018). Mitral regurgitation: anatomy is destiny. Eur J Cardio-Thorac Surg off J Eur Assoc Cardio-Thorac Surg.

[20] Malahfji M, Crudo V, Kaolawanich Y (2023). Influence of Cardiac Remodeling on clinical outcomes in patients with aortic regurgitation. J Am Coll Cardiol.

[21] Boyett Anderson JM, Hokanson JS (2022). Variation in management of paediatric isolated bicuspid aortic valve: current practice survey. Cardiol Young.

